# Scaffold-hopping identifies furano[2,3-*d*]pyrimidine amides as potent Notum inhibitors

**DOI:** 10.1016/j.bmcl.2019.126751

**Published:** 2020-02-01

**Authors:** Benjamin N. Atkinson, David Steadman, William Mahy, Yuguang Zhao, James Sipthorp, Elliott D. Bayle, Fredrik Svensson, George Papageorgiou, Fiona Jeganathan, Sarah Frew, Amy Monaghan, Magda Bictash, E. Yvonne Jones, Paul V. Fish

**Affiliations:** a Alzheimer’s Research UK UCL Drug Discovery Institute, University College London, Cruciform Building, Gower Street, London WC1E 6BT, UK; b Division of Structural Biology, Wellcome Centre for Human Genetics, University of Oxford, The Henry Wellcome Building for Genomic Medicine, Roosevelt Drive, Oxford OX3 7BN, UK; c The Francis Crick Institute, 1 Midland Road, Kings Cross, London NW1 1AT, UK

**Keywords:** Notum inhibitor, Wnt signaling, CNS penetration, Furano[2,3*-d*]pyrimidines, SBDD, Aβ, amyloid-beta, AD, Alzheimer’s disease, ADME, absorption distribution metabolism elimination, CNS, central nervous system, ER, efflux ratio, FP, fluorophosphonate, HBD, hydrogen bond donor, MLM, mouse liver microsomes, MPO, multiparameter optimization, OPTS, trisodium 8-octanoyloxypyrene-1,3,6-trisulfonate, P-gp, P-glycoprotein, SAR, structure activity relationship, SBDD, structure based drug design, TPSA, topological polar surface area, UPLC–MS, ultra performance liquid chromatography–mass spectrometer, HBTU, *O*-(1*H*-Benzotriazol-1-yl)-*N,N,N′,N′*-tetra-methyluronium hexafluorophosphate

## Abstract

The carboxylesterase Notum is a key negative regulator of the Wnt signaling pathway by mediating the depalmitoleoylation of Wnt proteins. Our objective was to discover potent small molecule inhibitors of Notum suitable for exploring the regulation of Wnt signaling in the central nervous system. Scaffold-hopping from thienopyrimidine acids **1** and **2**, supported by X-ray structure determination, identified 3-methylimidazolin-4-one amides **20**–**24** as potent inhibitors of Notum with activity across three orthogonal assay formats (biochemical, extra-cellular, occupancy). A preferred example **24** demonstrated good stability in mouse microsomes and plasma, and cell permeability in the MDCK-MDR1 assay albeit with modest P-gp mediated efflux. Pharmacokinetic studies with **24** were performed *in vivo* in mouse with single oral administration of **24** showing good plasma exposure and reasonable CNS penetration. We propose that **24** is a new chemical tool suitable for cellular studies to explore the fundamental biology of Notum.

## Graphical abstract

**Figure:**
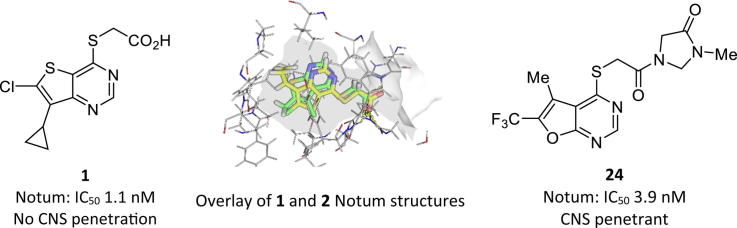

The Wnt signaling pathway regulates several aspects of brain development and function, and dysregulation of Wnt signaling has been implicated to play a role in neurodegenerative diseases such as Alzheimer’s disease (AD).^1^ Cognitive impairments, characteristic of AD, correlate closely with the loss of synapses and evidence suggests that excess amyloid-β (Aβ) causes synapse dysfunction by impairing synapse maintenance, at least in part, through causing dysfunction of Wnt signaling.^2^, ^3^ Compromised Wnt signaling may also be associated with AD through loss of blood-brain barrier (BBB) integrity^4^ and Aβ generation through β-secretase (BACE1) expression.^5^

Signal transduction by Wnt proteins is tightly regulated by a range of mechanisms including post translational modifications. For example, *O*-palmitoleoylation of Wnt proteins is required for efficient binding to Frizzled (Fzd) receptors and the subsequent signal transduction.^6^ The carboxylesterase Notum is a key negative regulator of the Wnt signaling pathway by specifically mediating the *O*-depalmitoleoylation of Wnt proteins.^7^, ^8^ The role of Notum in the mammalian central nervous system (CNS) has yet to be established although *Notum* is expressed and upregulated in endothelial cells in the hippocampus of APPPS1 mice and AD patients compared to control.^9^ In a disease setting, it follows that inhibition of Notum could restore Wnt signaling with potential benefit in disease where Wnt deficiency is an underlying cause.

**Figure:**
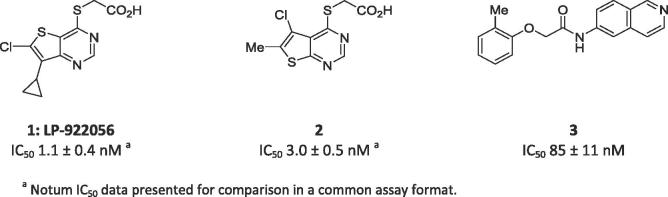

The search for Notum inhibitors has identified acids **1** and **2** which have shown utility in mouse models of bone growth and found to be increase cortical bone thickness.^10^, ^11^ Although **1** demonstrates good oral bioavailability, recent pharmacokinetic studies in mouse showed CNS penetration of **1** is very low with brain:plasma concentration ratio of just 0.01.^12^ Additional compounds include irreversible inhibitor **ABC99** used to show the role of Notum in the regeneration of aged intestinal epithelium,^13^, ^14^ and phenoxyacetamide **3** identified through optimisation of an X-ray fragment screening hit.^15^ However, it is unlikely that these compounds will be suitable for *in vivo* studies where CNS penetration is an essential requirement. Hence, our objective was to discover potent small molecule inhibitors of Notum suitable for exploring the regulation of Wnt signaling in the CNS.

In order to identify new small molecule inhibitors of Notum, we elected to explore if **1** and **2** could be modified to deliver a CNS penetrant tool by capping off the acid as an amide. However, prior art had established that similar carboxamides exhibited poor metabolic stability.^10^ Our initial investigations into amide derivatives of **1** somewhat confirmed this result but also showed that judicious choice of the amine partner could significantly improve metabolic stability as measured in liver microsomes.^12^

At the outset, we wished to use structure based drug design (SBDD) to accelerate our progress towards the discovery of potent inhibitors by effective binding with Notum. Crystals of *C*-terminal his-tagged Notum(Ser81-Thr451 Cys330Ser) were soaked with acids **1** and **2**, and the crystal structures solved to elucidate their inhibitor binding modes (Fig. 1). Notum has a well-defined, large (ca. 380 Å^3^), hydrophobic active-site pocket adjacent to the catalytic triad (Ser232, His389, Asp340) that accommodates the palmitoleate group of Wnt (PDB:4UZQ).^7^ Both **1** and **2** place the thienopyrimidine group into this pocket with the acid forming the only polar interactions through a network of H-bonds to the backbone with Trp128, Gly127 and Ala233, and also a H-bond to the sidechain of His389 (Fig. S1). The position of the thiophene ring differs slightly between **1** and **2** to accommodate the substituents which sit on opposite sides of the inhibitor, but the remainder of the molecules adopted a similar position in the pocket. Overlays of the structures of **1** and **2** with *O*-palmitoleoyl serine show all three structures effectively fill this pocket (Fig. S2). From a design perspective, these structures show significant solvent exposed space at the mouth of the palmitoleate pocket to accommodate a suitable group as an amide derivative of **1** and **2**.

**Fig. 1:**
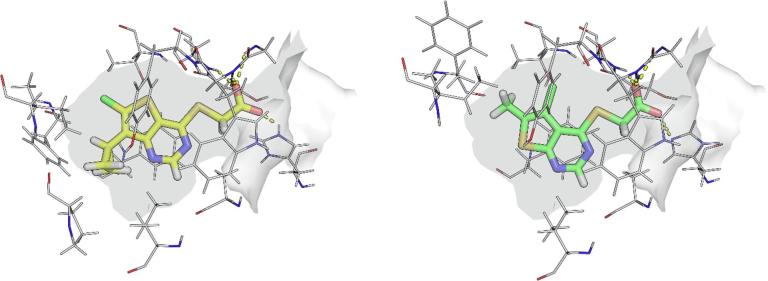
Crystal structures of **1** (yellow) and **2** (green) with the surface of the Notum palmitoleoyl binding pocket outlined (grey). Binding site residues shown within 3 Å of their respective ligands. Key hydrogen bond interactions are shown as dashed lines. Water molecules have been removed for clarity. Atomic coordinates have been deposited in the Protein Data Bank (PDB). PDB ID codes: **1**: 6T2K; **2**: 6T2H. (For interpretation of the references to colour in this figure legend, the reader is referred to the web version of this article.)

The SARs were initially directed at exploring two principle areas of the structure: (1) the amide group (**4**, **5**) (Table 1, Table 2 and S1); and (2) the pyrimidine heterocyclic group that binds in the palmitoleate pocket (**6**–**19**) (Table 3). Combinations of preferred amides and heterocycles were then prepared (**20**–**24**) (Table 4). Minimising compound lipiphilicity is a well-established approach to improve overall drug-like properties, although this would need to be tempered by the requirement for CNS penetration.^16^ Target compounds were designed to have molecular and physicochemical properties consistent with CNS drug-like space and we used the CNS MPO score to aide our design.^17^ In general, target compounds **4**, **5** and **20**–**24** all demonstrated CNS MPO scores >4.0 and had cLogP values in the range 1.5–3.2.

**Table 1:** Comparison of thieno[3,2-*d*]pyrimidine amides **4**^a^ with thieno[2,3-*d*]pyrimidine amides **5**.

**Figure:**
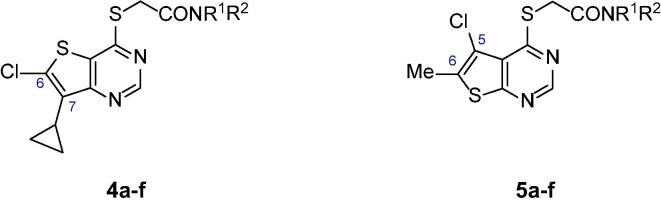

NR^1^R^2^	Compound	Notum^b^ IC_50_ (nM)	MLM^c^ Cl_i_ (μL/min/mg)	MDCK-MDR1^c^ AB/BA *P*_app_ (×10^−6^ cm/s) and efflux ratio (ER)	
–NMe_2_	**4a**	7.5 ± 12.4			
	**5a**	15 ± 6	360		
	**4b**	91 ± 67			
	**5b**	220 ± 12			
	**4c**	18 ± 8.7	>500	40/38	0.95
	**5c**	69 ± 10			
	**4d**	7.1 ± 4.1	24	7.9/65	8.2
	**5d**	5.8 ± 4.0	19	12/66	5.5
	**4e**	1.5 ± 0.1	19	3.8/14	3.7
	**5e**	2.7 ± 0.5	65	14/82	5.9
	**4f**	1.1 ± 0.3	29	0.95/93	98
	**5f**	3.2 ± 0.1	13	0.6/56	93

a See Ref. 12.

b All values are geometric mean ± s.d. of n = 2–6 experiments quoted to 2 s.f. Differences of <2-fold should not be considered significant. For details of the assay protocol, see reference 15.

c MLM, MDCK-MDR1 and additional in vitro ADME studies reported in this work were independently performed by GVK Biosciences (Hyderabad, India. https://www.gvkbio.com/discovery-services/biology-services/dmpk-services/) or Cyprotex (Macclesfield, UK. https://www.cyprotex.com/admepk).

**Table 2:** Notum inhibition, MLM stability and MDCK-MDR1 cell permeability of thieno[2,3-*d*]pyrimidine amides **5**.^a^

**Figure:**
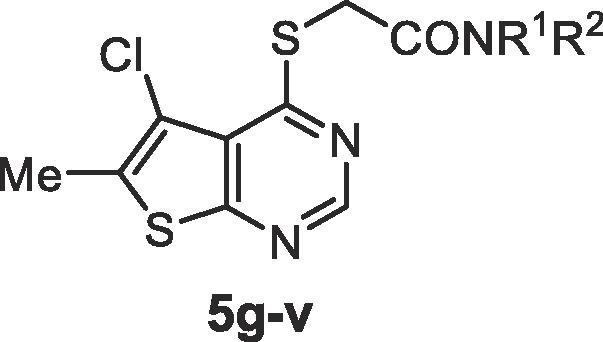

NR^1^R^2^	Compound	Notum IC_50_ (nM)	MLM Cl_i_ (μL/min/mg)	MDCK-MDR1 AB/BA *P*_app_ (×10^−6^ cm/s) and efflux ratio (ER)	
	**5g**	6.2 ± 0.5	33	23/62	2.7
	**5h**	4.2 ± 0.4	100	1.1/37	34
	**5i**	33 ± 5			
	**5j**	450 ± 200			
	**5k**	20 ± 4	63	7/51	7.3
	**5l**	2.4 ± 0.4	25	36/42	1.2
	**5m**	2.6 ± 0.1	43	35/51	1.5
	**5n**	3.8 ± 1.3	49		
	**5o**	3.9 ± 0.4	140		
	**5p**	16 ± 2	15	0.5/51	>100
	**5q**	23 ± 5	38	0.8/65	81
	**5r**	27 ± 3	53	0.8/78	97
	**5s**	38 ± 3	93	15/85	5.7
	**5t**	15 ± 3	53	19/49	2.6
	**5u**	11 ± 6	37	17/44	2.6
	**5v**	18 ± 5	140	15/27	1.8

a See footnotes Table 1.

**Table 3:** Notum inhibition of thieno- (**6**–**12**), pyrrolo- (**13**–**16**), pyrazolo- (**17**) and furanopyrimidine acids (**18**–**19**).

**Figure:**
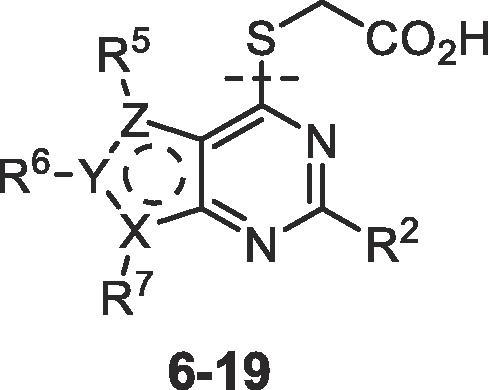

Compound	Notum IC_50_ (nM)^a^	Compound	Notum IC_50_ (nM)^a^
	790 ± 70^b^		77,000 ± 28,000^b^
	5.8 ± 0.5 b		20,000 ± 3,900
	2,100 ± 330		15,000 ± 1,700
	1.0 ± 0.4		50,000 ± 6,500
	3.9 ± 0.7		1,100 ± 240
	25 ± 4		50 ± 14^b^
	8.1 (n = 1)		3.2 ± 0.5

a See footnotes Table 1.

b Notum IC_50_ values for published compounds **6**, **7**, **13**, **18** are presented for comparison in a common assay format and to define SAR relationships: **6**, 152 nM; **7**, 2 nM; **13**, 15,000 nM; **18**, not disclosed. See, Ref. 10.

**Table 4:** Notum inhibition, MLM stability and MDCK-MDR1 cell permeability of preferred 3-methylimidazolin-4-one amides **20**–**24**.

**Figure:**
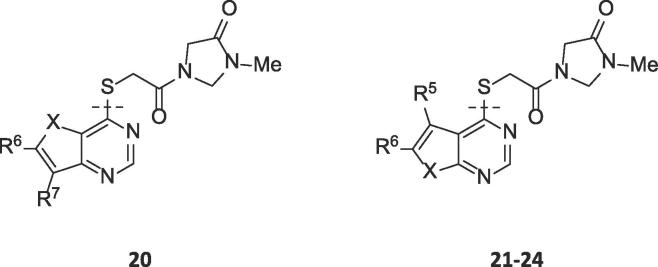

Het	Compound	Notum IC_50_ (nM)^a^ EC_50_ (nM)^b^	MLM Cl_i_ (μL/min/mg)	MDCK-MDR1 AB/BA *P*_app_ (×10^−6^ cm/s) and efflux ratio (ER)	
	**20**	1.5 ± 0.2 110 ± 67	45	5.9/12	2.0
	**21**	9.4 ± 1.5 1300 ± 170	31	21/45	2.1
	**22**	1.6 ± 0.1 210 ± 130	27	31/35	1.1
	**23**	7.0 ± 2.5 170 ± 31	24		
	**24**	3.9 ± 0.8 220 ± 64	6.9	23/56	2.4

a Notum OPTS assay, see footnotes Table 1.

b Notum TCF-LEF assay. All values are geometric mean ± s.d. of n = 3–4 experiments quoted to 2 s.f. For details of the assay protocol see Ref. 15.

Target compounds **4**–**24** were prepared in two phases: advanced intermediates 4-chloropyrimidines **25** were either purchased or prepared using a customised synthesis (see Supplementary material, Schemes S1–S16) and then a short sequence was used to prepare **4**–**24** from **25** (Scheme 1). Nucleophilic displacement of the C4-Cl of **25** by methyl thioglycolate gave ester **26** which was hydrolysed with NaOH to afford the corresponding acid (**1**, **2**, **6**–**19**). Finally, activation of the acid with HBTU and subsequent reaction with the amine (HNR^1^R^2^) afforded the amide (**4**, **5**, **20**–**24**).

**Scheme 1:**
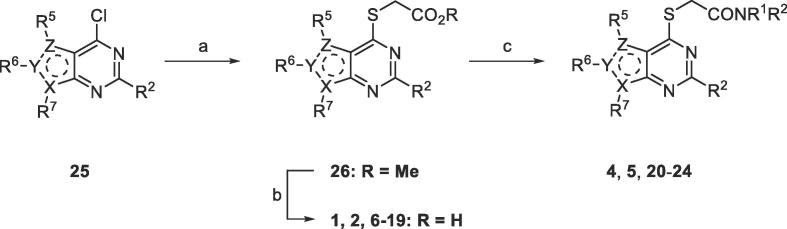
Preparation of acids **1**, **2**, **6**–**19** and amides **4**, **5**, **20**–**24**. Representative reagents and conditions: (a) HSCH_2_CO_2_Me (1.2 equiv.), NEt_3_ (2.1 equiv.), MeOH, 0 °C to rt; (b) NaOH (1 M) (2 equiv.), THF, 0 °C, then HCl (1 M), 0 °C; (c) HBTU (1.1 equiv.), iPr_2_NEt (2.5 equiv.), DMF, rt, 15 min; then amine (HNR^1^R^2^) (1.05 equiv.).

Inhibition of Notum carboxylesterase activity of **4**–**24** (Table 1, Table 2, Table 3, Table 4) was routinely measured in a biochemical assay where test compounds were incubated with Notum(81–451 Cys330Ser) and trisodium 8-octanoyloxypyrene-1,3,6-trisulfonate (OPTS) as the substrate for 1 h and fluorescence recorded.^15^ Compounds were then assessed for metabolic stability in mouse liver microsomes (MLM) and for cell permeability by measuring transit performance across a MDCK-MDR1 monolayer. Selected compounds were screened for inhibition of Notum activity in a Wnt/β-catenin signaling pathway TCF/LEF Reporter (Luciferase) HEK293 cell line and Notum occupancy in a FP-biotin competition assay.^15^, ^18^

Initial SAR studies with amides **4** and **5** (derived from **1** and **2** resp.) suggested that the Notum activity was largely driven by the heterocycle binding in the palmitoleate pocket with the amide moiety offering minimal contribution, although poor choice of amine partner could disrupt the binding; this is consistent with X-ray structures and docking studies (Table 1).^19^, ^20^ From this set of matched pairs, three amide series **4d**/**5d**, **4e**/**5e** and **4f**/**5f** emerged as having potent Notum inhibition (IC_50_ < 10 nM), moderate MLM stability and cell permeability although they were all substrates for P-gp mediated efflux to some degree. The challenge then became to retain Notum inhibition activity, further improve metabolic stability whilst developing cell permeability without efflux.

A wider range of amides around **5d-f** were then prepared in the thieno[2,3-*d*]pyrimidine series **5** as this template offered the advantage of slightly lower lipophilicity when compared to **4** (**5** vs **4**, ΔcLogP = −0.5) (Tables 2 and S1). One approach to reduce P-gp mediated efflux is to remove HBD or, if the HBD is essential for binding to the primary target, to partially mask the HBD group by placing a flanking group in close proximity. *N*-Alkylation of the piperazin-2-one **5d** with either a Me (**5g**) or Et (**5h**) group retained potency although impact on efflux was inconsistent. *C*-Alkylation of **5d** at the α-position with one or two Me groups reduced potency (**5i**, **5j**) and combining these two modifications into ring gave **5k** which was inferior to **5d** in all aspects. *N*-Methylation of imidazolidin-4-one **5e** proved to more beneficial with **5l** showing potent activity, improved MLM stability and high cell permeability with minimal efflux (ER 1.2). *N*-Substitution with larger alkyl groups such as Et (**5m**), cPr (**5n**) and CH_2_CF_3_ (**5o**) retained potent Notum inhibition but eroded MLM stability, and the instability tracked with increased compound lipophilicity.

Modifications to the triazolo[4,3-*a*]pyrazine amide **5f** proved to be detrimental. Alkylation of the available C3 position of the triazole ring with small lipophilic groups (**5p-s**: Me, Et, cPr, CF_3_) proved to be progressively detrimental to activity. Switching to the triazole isomer triazolo[1,5-*a*]pyrazine amides **5t-v** also reduced potency although substitution at C2 was tolerated but offered little advantage. These triazolopyrazine amides **5p-v** were at least 3-fold weaker than **5f** and failed to improve MLM or the efflux ratio.

At this point, mouse pharmacokinetic data for **5l** was generated *in vivo* to determine the extent of plasma exposure and to check the correlation of the *in vitro* ADME data with *in vivo* outcomes. Imidazolidin-4-one **5l** (cLogP 2.6; LogD_7.4_ 1.8) was selected as a representative example from this set as it combined good aqueous solubility (77 μg/mL; 215 μM) and cell permeability with moderate microsomal stability. Following single oral dose (p.o.) of 10 mg/kg, plasma exposure for **5l** was low (C_max_ 120 ng/mL; AUC_(0→inf)_ 70 ng.h/mL) which we attribute to high clearance and highlighted the need to further improve metabolic stability (Fig. S4).

The next phase of SAR was to explore the pyrimidine heterocyclic group that binds in the palmitoleate pocket (Table 3). This series of SARs was performed with the carboxylic acids with a view to introducing the amide group once preferred heterocycles had been identified. Thienopyrimidines **1** and **2** are potent inhibitors and so a range of alternative substituents on the thiophene ring (**6**–**12**) were investigated. Substituents were selected to optimise binding interactions with Notum and reduce overall lipophilicity through removal of lipophilic groups and/or introduction of polar groups. Deletion of the 5-Cl from **2** to give **6** (5-H) resulted in a significant drop in activity whereas direct replacement of 5-Cl with a 5-Me **7** retained activity. Further modification of the **7** scaffold by the addition of a 2-Me group (**8**) led to a dramatic decrease in potency and so substitution at C2 was not investigated further. Introduction of a CF_3_ group at either the 5- or 6-positions (**9**, **10** resp.) proved to be beneficial whereas the application of a 6-CN as a non-traditional bioisostere for a halogen (**11**, **12**) was detrimental to activity on this occasion.^21^

Alternative fused 6,5-ring systems (**13**–**19**) were also explored with the objective of replacing the thiophene with a more polar N or O containing heterocycle. Pyrrolopyrimidine **13** was a weak inhibitor although activity was improved when combined with substituents at either the 5- or 7- positions (**14**, **15**). A 7-Bn group (**16**) could be accommodated but there was no significant improvement over 7-H (**13**), and this was at a significant penalty in added lipophilicity. Pyrazolopyrimidine **17** proved to be the most active inhibitor from these N heterocycles although still 1000-fold weaker than **1**. In contrast, the furano[2,3-*d*]pyrimidines proved to be more successful when combined with optimal substituents. 5,6-Dimethyl furan **18** was 10-fold weaker than the corresponding thiophene analogue (**18** vs **7**) but replacement of the 6-Me of **18** with a 6-CF_3_ gave **19** which restored potent Notum inhibition activity in this more polar template (**19** vs **9**; ΔcLogP = −0.6).

The strategy of combining the superior acid heterocycles (**1**, **7**, **9**, **10**, **19**) with the preferred 3-methylimidazolin-4-one amine produced amides **20**–**24** all with potent Notum inhibition (IC_50_ < 10 nM) in the biochemical OPTS assay (Table 4). In general, the Notum inhibition activity of these amides tracked closely to the activity of their corresponding acid, and with the same rank order, again suggesting the amide moiety offered minimal contribution (or disruption) to the binding with Notum.

These inhibitors **20**–**24** were screened in the cell-based TCF/LEF reporter gene (Luciferase) assay to assess their ability to restore Wnt/β-catenin signaling when activated by exogenous rWNT3a (100 ng/mL) in the presence of Notum (500 ng/mL) (Table 4). Compounds **20**, **22**–**24** all showed an effective activation of Wnt signaling (EC_50_ < 250 nM) in this model system through inhibition of Notum. In contrast, **21** showed only modest activation of Wnt signaling.

Evaluation of **20**–**24** in MLM showed **20**–**23** to have moderate metabolic stability and offered no significant advantage over **5l**. Only **24** demonstrated high metabolic stability in MLM with the potential advantage of low metabolic clearance *in vivo*. Furthermore, **24** was stable in mouse plasma with no degradation observed after 120 min and did not inhibit CYP450 enzymes (Table 5). Compound **24** displayed a modest efflux ratio (ER = 2.4) in the MDCK-MDR1 permeability assay which suggests some recognition by P-gp mediated efflux transport. However, the ER for **24** was perceived to be within acceptable limits based on established precedent.^22^

**Table 5:** Summary of physicochemical and molecular properties, Notum inhibition and ADME data for **24**.

	**24**
*Physicochemical and molecular properties*	
mol wt	374
cLogP	2.1
LogD_7.4_	1.6
TPSA (Å^2^)	74.6
CNS MPO	5.9

*Notum inhibition*^a^	
OPTS, IC_50_ (nM)	3.9 ± 0.8 (n = 4)
TCF-LEF, EC_50_ (nM)	220 ± 64 (n = 3)

*ADME profile*^b^	
Aq. solubility (μg/mL)/(μM)	45/120
Mouse plasma protein binding (PPB) (%)	78.1
Mouse brain binding (%)	84.4
MLM, Cl_i_ (μL/min/mg protein)	6.9
Mouse plasma stability, % remaining at 120 min (%)	110
CYP1A2 inhibition, IC_50_ (μM)	>30
CYP2B6 inhibition, IC_50_ (μM)	>30
CYP2C9 inhibition, IC_50_ (μM)	>30
CYP2D6 inhibition, IC_50_ (μM)	>30
CYP3A4 inhibition, IC_50_ (μM)	>30
MDCK-MDR1, AB/BA *P_app_* (×10^−6^ cms^−1^)	23/56
MDCK-MDR1, efflux ratio (ER)	2.4

a See footnotes Table 4.

b *In vitro* ADME studies reported in this work were performed by GVK Biosciences (Hyderabad, India).

Representative inhibitors **5l** and **24** were tested in a Notum occupancy assay using FP-biotin,^18^ a covalent serine hydrolase activity-based probe, whereby labelling of Ser232 of Notum with FP-biotin can be blocked by an inhibitor occupying the active site of Notum (Figs. 2 and S3). As **5l** and **24** are reversible, high affinity inhibitors of Notum, it proved necessary to adapt our reported protocol^15^ to ensure we were in the dynamic range of labelling by FP-biotin in the presence of **5l** and **24** so that *relative* potencies could be determined. Compounds **1** and **3** have been evaluated under both assay conditions and are used as standards to help bridge results from these studies.^15^ Both **5l** and **24** showed an ability to prevent labelling by FP-biotin, confirming they competitively bind to Notum, with potency equivalent to **1**.

**Fig. 2:**
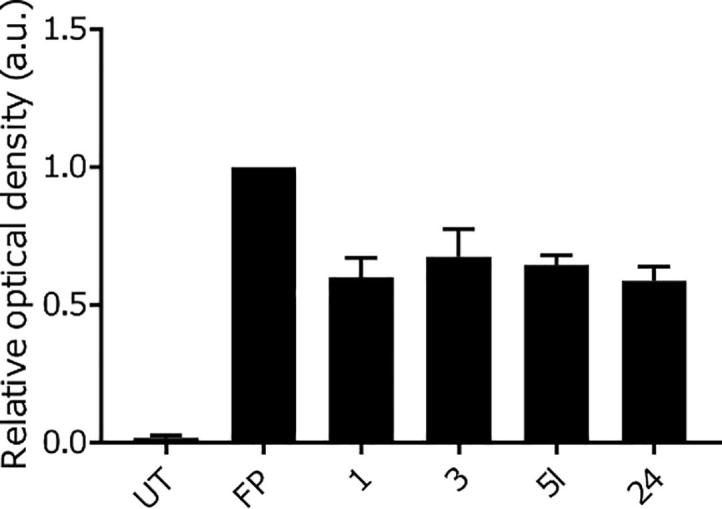
Notum activity-based occupancy assay was performed with FP-biotin (FP) (2 μM) and test compounds **1**, **3**, **5l** and **24** (3 μM) for 30 min in conditioned media from HEK293S cells stably transfected with a Notum lentiviral construct. Relative occupancy was calculated by optical density of the fluorescent band, generated by streptavidin linked fluorophore to detect the level of biotinylation of Notum using Image Studio Lite 5.2, compared to the control-treated sample which was set to 1. N = 2 with S.D. UT, untreated.

Hence, on balance, **24** emerged as having a superior profile from this set and was selected for further evaluation in mouse pharmacokinetic studies.

Pharmacokinetic data for **24** was generated *in vivo* in mouse to evaluate plasma exposure and CNS penetration (Table 6; Fig. S4). Following single oral dose (p.o.) of 10 mg/kg, plasma exposure achieved a C_max_[plasma] ≈ 2.2 μM (free drug) which significantly exceeds the Notum EC_50_ from the cell-based TCF/LEF assay. However, the plasma half-life was moderate which was somewhat unexpected based on the *in vitro* MLM and mouse plasma stability data. Compound **24** demonstrates reasonable CNS penetration with a brain:plasma concentration ratio of 0.29 based on AUC_(0→inf)_. The incomplete CNS penetration was probably due to some element of P-gp efflux transport recognition as evidenced by the ER in the MDCK-MDR1 cell line. The combination of incomplete CNS penetration along with preferential binding to brain tissue resulted in moderate brain exposure of C_max_ [brain] ≈ 0.5 μM (free drug) but this still exceeds the Notum EC_50_ at this dose.

**Table 6:** Mouse pharmacokinetic data for **24**; oral (p.o.) dose at 10 mg/kg.^a^

PK Parameter	Plasma	Brain
T_1/2_	0.6 h	0.8 h
T_max_	0.5 h	0.5 h
C_max_	3850 ng/mL	1210 ng/g
AUC_(0-t)_	5390 ng.h/mL	1550 ng.h/g
AUC_(0-inf)_	5490 ng.h/mL	1610 ng.h/g

a Male C57BL6 mice; suspension formulation in 0.1% Tween80 in water; n = 3 per time point; terminal blood and brain levels measured at seven time points: 0.17, 0.50, 1, 2, 4, 8 and 24 h. All animals were healthy throughout the study period.

Hence, **24** has *potential* utility in mouse models of disease under carefully designed experimental protocols where the required site of action, route of administration, dose and duration of action requirements are understood; i.e. the pharmacokinetic-pharmacodynamic relationship is to be established.

In summary, scaffold-hopping from thienopyrimidine acids **1** and **2**, supported by X-ray structure determination, identified 3-methylimidazolin-4-one amides **20**–**24** as potent inhibitors of Notum with activity across three orthogonal assay formats. A preferred example **24** demonstrated good stability in MLM and mouse plasma, and cell permeability in the MDCK-MDR1 assay albeit with modest P-gp mediated efflux. PK studies with **24** were performed *in vivo* in mouse with single oral administration of **24** showing good plasma exposure and reasonable CNS penetration. We propose that **24**^23^ is a new chemical tool suitable for cellular studies to explore the fundamental biology of Notum. Amide **24** has complementary properties to CNS excluded acid **1** and irreversible inhibitor **ABC99**, and so represents a valuable addition to the Notum inhibitor chemical toolbox.

## Declaration of Competing Interest

The authors declare that they have no known competing financial interests or personal relationships that could have appeared to influence the work reported in this paper.

## Appendix A Supplementary data

Supplementary data 1 (1) Interaction maps of **1** and **2** in the Notum binding pocket; (2) Overlays of compounds **1**, **2** and *O*-palmitoleoyl serine in the Notum binding pocket; (3) Western blots used to generate activity-based probe occupancy assay data; (4) Mouse PK data for **5l** and **24**; (5) Notum inhibition, MLM stability and MDCK-MDR1 cell permeability of additional thieno[2,3-*d*]pyrimidines amides **5w-5uu**; (6) Synthetic schemes for the preparation of 4-chloropyrimidines **25**.
