# Supplementary material for: Scaffold-hopping identifies furano[2,3-d]pyrimidine amides as potent Notum inhibitors
Source: Bioorg Med Chem Lett. 2020 Feb 1;30(3):126751. doi: 10.1016/j.bmcl.2019.126751 (PMC6961116; doi:10.1016/j.bmcl.2019.126751)
Supplement: Supplementary data 1 — (1) Interaction maps of 1 and 2 in the Notum binding pocket; (2) Overlays of compounds 1, 2 and O-palmitoleoyl serine in the Notum binding pocket; (3) Western blots used to generate activity-based probe occupancy assay data; (4) Mouse PK data for 5l and 24; (5) Notum inhibition, MLM stability and MDCK-MDR1 cell permeability of additional thieno[2,3-d]pyrimidines amides 5w-5uu; (6) Synthetic schemes for the preparation of 4-chloropyrimidines 25. [file mmc1.docx]

**Scaffold-hopping identifies furano[2,3-*d*]pyrimidine amides as potent Notum inhibitors**

Benjamin N. Atkinson,^1^ David Steadman,^1^ William Mahy,^1^ Yuguang Zhao,^2^ James Sipthorp,^1,3^ Elliott D. Bayle, ^1,3^ Fredrik Svensson,^1,3^ George Papageorgiou,^3^ Fiona Jeganathan,^1^ Sarah Frew,^1^ Amy Monaghan,^1^ Magda Bictash,^1^ E. Yvonne Jones,^2^ Paul V. Fish.^1,3,^ *

^1^ Alzheimer’s Research UK UCL Drug Discovery Institute, University College London, Cruciform Building, Gower Street, London, WC1E 6BT, U.K.

^2^ Division of Structural Biology, Wellcome Centre for Human Genetics, University of Oxford, The Henry Wellcome Building for Genomic Medicine, Roosevelt Drive, Oxford, OX3 7BN, U.K.

^3^ The Francis Crick Institute, 1 Midland Road, Kings Cross, London NW1 1AT, U.K.

**SUPPORTING MATERIAL**

**Table of contents:**

Page S2 **Figure S1**. Interaction maps of **1** and **2** in the Notum binding pocket.

Page S3 **Figure S2.** Overlays of compounds **1**, **2** and *O*-palmitoleoyl serine in the Notum binding pocket.

Page S5 **Figure S3**. Western blots used to generate activity-based probe occupancy assay data.

Page S6 **Figure S4**. Mouse PK for compounds **5l** and **24**.

Page S7 **Table S1**. Notum inhibition, MLM stability and MDCK-MDR1 cell permeability of thieno[2,3-*d*]pyrimidine amides **5w-5uu**.

Page S10 **Schemes S1-S16**. Synthetic schemes for the preparation of 4-chloropyrimidines **25**.

Page S14 **SM References**

**Figure S1: Interaction maps**

**
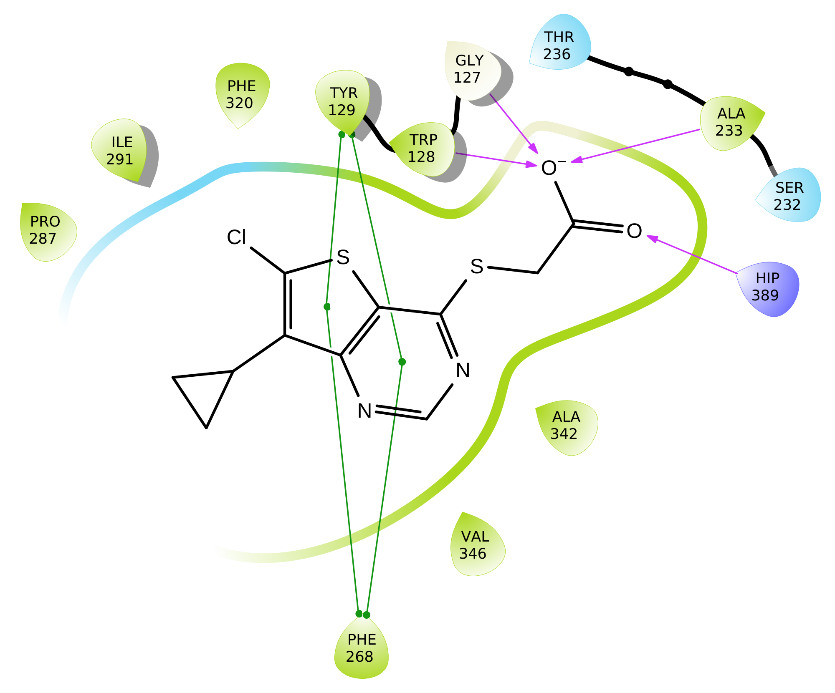
**

**Figure S1A.** Interaction map for **1** showing the acid forming the only polar interactions through a network on H-bonds to the backbone with Trp128, Gly127 and Ala233, and also a H-bond to the sidechain of His389. The the thieno[3,2-*d*]pyrimidine group resides in the palmitoleate pocket with pi-pi interaction between Tyr129 and Phe268. Hip389 is the (putative) protonated form of His389.

**
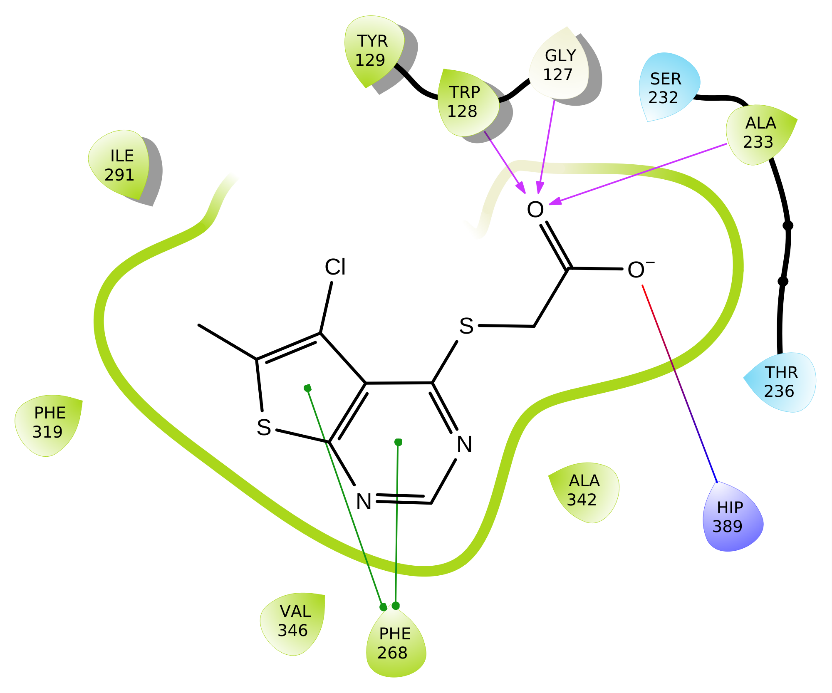
**

**Figure S1B.** Interaction map for **2** showing the acid forming the only polar interactions through a network on H-bonds to the backbone with Trp128, Gly127 and Ala233, and also a H-bond to the sidechain of His389. The the thieno[2,3-*d*]pyrimidine group resides in the palmitoleate pocket with a pi-pi interaction with Phe268. Hip389 is the (putative) protonated form of His389.

**Figure S2: Overlays of compounds 1, 2 and *O*-palmitoleoyl serine in the Notum binding pocket.**


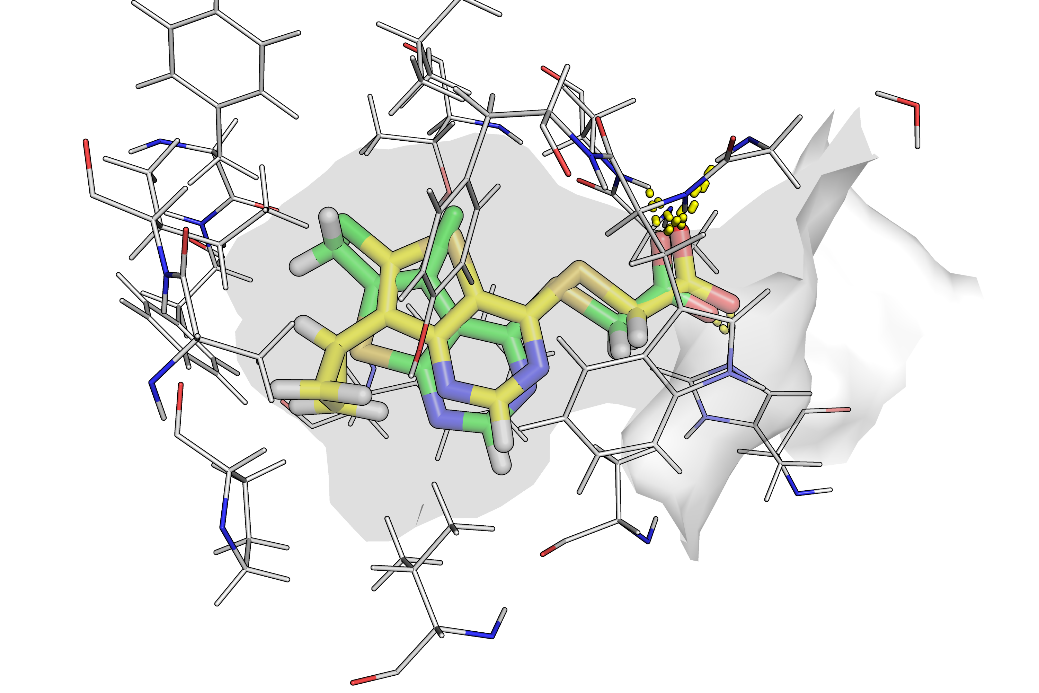


**Figure S2A.** Overlay of **1** (yellow) and **2** (green) in the PDB 4UZQ crystal structure with the surface of the binding pocket outlined. Key hydrogen bond interactions are shown as dashed lines. Water molecules have been removed for clarity.


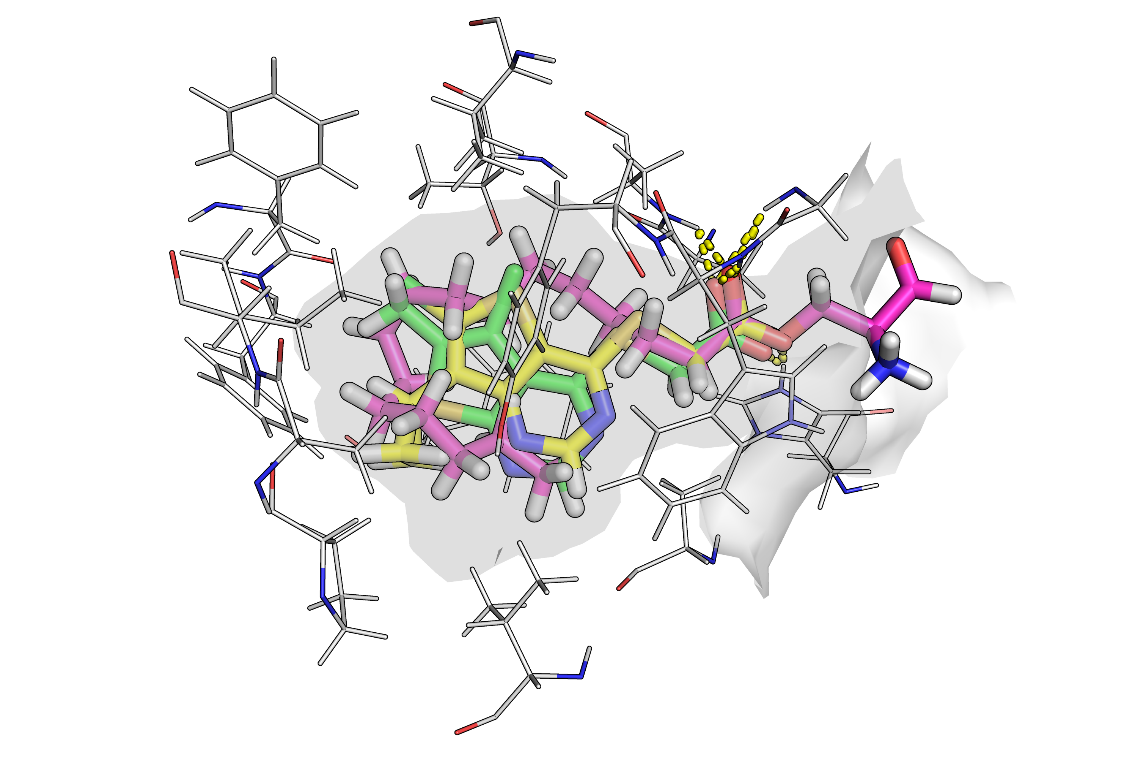


**Figure S2B.** Overlay of **1** (yellow), **2** (green) and *O*-palmitoleoyl serine in the PDB 4UZQ crystal structure with the surface of the binding pocket outlined. Key hydrogen bond interactions are shown as dashed lines. Water molecules have been removed for clarity.


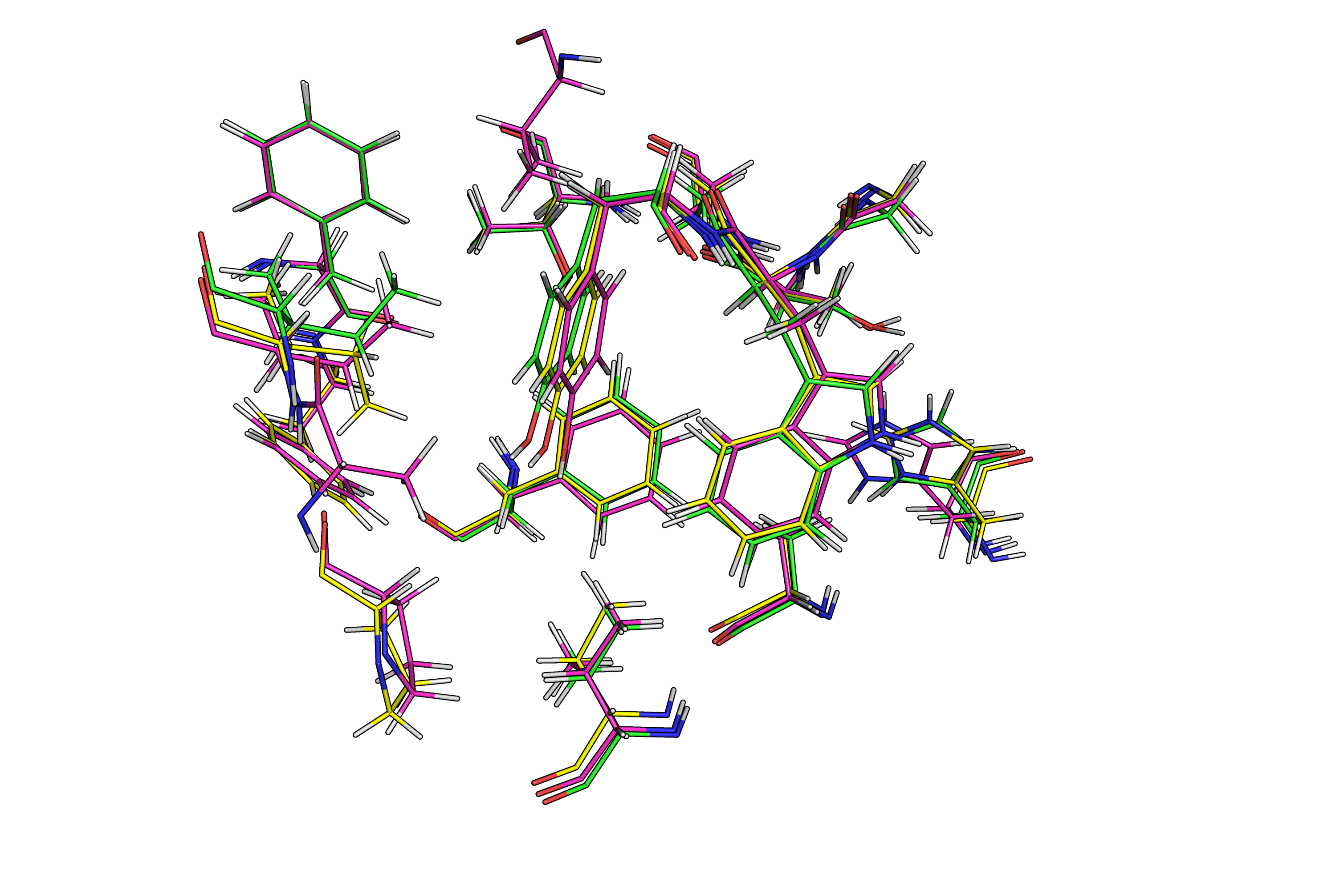


**Figure S2C.** Overlay of binding site residues within 3Å of their respective ligands. The overall geometry of the active site is well preserved across the different structures. **1** (yellow), **2** (green), and *O*-palmitoleoyl serine (magenta). Ligands have been removed for clarity.

**Figure S3: Western blots used to generate activity-based probe occupancy assay data.**

**
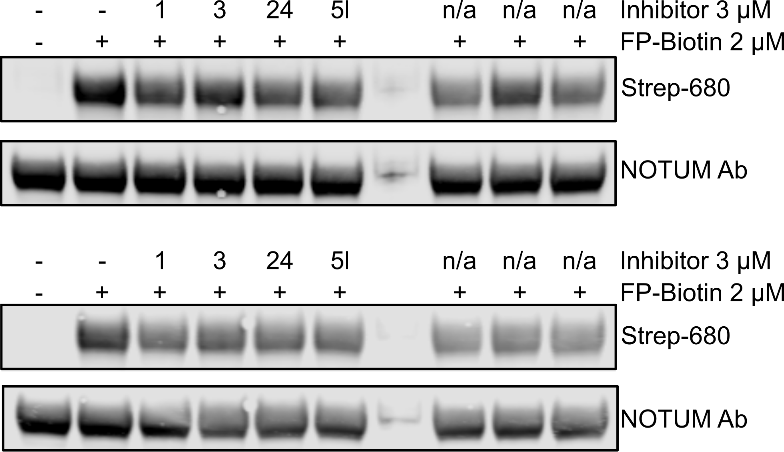
**

The general experimental procedure for this assay was used as previously disclosed by Atkinson *et al*.,^1^ but with the following modifications. Compounds were pre-incubated with Notum at a concentration of 3 μM prior to the addition of FP-biotin. Additionally, FP-biotin was incubated with the concentrates for 30 mins prior to quenching the reaction with 4X LDS sample buffer, 10X reducing agent and then heating samples to 75 °C for 10 mins.

**Figure S4:**

**A. Mean (± s.d.; n = 3) plasma and brain concentration vs time profile of compound 5l after oral administration (10 mg/kg) to male C57BL/6 mice.**

**B. Mean (± s.d.; n = 3) plasma and brain concentration vs time profile of compound 24 after oral administration (10 mg/kg) to male C57BL/6 mice.**

*Animal Testing Declaration: In vivo* mouse pharmacokinetic data was generated at GVK Biosciences, Hyderabad, India. GVK Biosciences is accredited by AAALAC and OLAW for the ethical treatment of animals. All the animal experiments are conducted in accordance with IAEC approved protocols. See: <https://www.gvkbio.com/biology-services/in-vivo-pharmacology/>

**Table S1:**

Notum inhibition, MLM stability and MDCK-MDR1 cell permeability of thieno[2,3-*d*]pyrimidine amides **5**.

Additional thieno[2,3-*d*]pyrimidine amides **5w-5uu** were prepared by the general methods as described and data presented here for a more complete analysis of the SARs.

|  |
| --- |
| **5w-5uu** |

| **NR^1^R^2^** | **Compound** | **Notum** ^a^  IC_50_ (nM) | **MLM** ^b^  Cl_i_ (μL/min/mg) | **MDCK-MDR1** ^b^  AB/BA *P*_app_ (x10^6^ cm/s) and efflux ratio (ER) | |
| --- | --- | --- | --- | --- | --- |
|  | **5w** | 106 ± 11 |  |  |  |
|  | **5x** | 33 ± 2 |  |  |  |
|  | **5y** | 290 ± 90 |  |  |  |
|  | **5z** | 25 ± 5 |  |  |  |
|  | **5aa** | 22 ± 3 |  |  |  |
|  | **5bb** | 1900 ± 850 |  |  |  |
|  | **5cc** | 24 ± 1 |  |  |  |
|  | **5dd** | 1.7 ± 0.1 | 300 |  |  |
|  | **5ee** | 2.7 ± 0.9 | 130 |  |  |
|  | **5ff** | 20 ± 11 |  |  |  |
|  | **5gg** | 88 ± 10 |  |  |  |
|  | **5hh** | 18 ± 7 | 100 |  |  |
|  | **5ii** | 15 ± 2 | 300 | 18/46 | 2.6 |
|  | **5jj** | 44 ± 8 |  |  |  |
|  | **5kk** | 61 ± 24 | 300 |  |  |
|  | **5ll** | 26 ± 6 |  |  |  |
|  | **5mm** | 33 ± 5 |  |  |  |
|  | **5nn** | 17 ± 5 | 3.7 | 0.95/61 | 65 |
|  | **5oo** | 62 ± 20 | 8.1 | 3.3/80 | 24 |
|  | **5pp** | 35 ± 6 | 60 | 0.8/69 | 88 |
|  | **5qq** | 24 ± 4 | 44 | 6.4/81 | 13 |
|  | **5rr** | 20 ± 6 |  |  |  |
|  | **5ss** | 7.8 ± 1.4 | 323 |  |  |
|  | **5tt** | 21 ± 4 | >500 |  |  |
|  | **5uu** | 3.2 ± 0.4 | 220 |  |  |

^a^ All values are geometric mean ± s.d. of n = 2-4 experiments quoted to 2 s.f. Differences of <2-fold should not be considered significant. For details of the assay protocol, see main text and references therein.

^b^ MLM and MDCK-MDR1 reported in this work were independently performed by GVK Biosciences (Hyderabad, India. <https://www.gvkbio.com/discovery-services/biology-services/dmpk-services/>) or Cyprotex (Macclesfield, UK. <https://www.cyprotex.com/admepk>).

**Scheme S1: Synthesis of 4,6-dichloro-7-cyclopropylthieno[3,2-d]pyrimidine (towards 1).**

4,6-Dichloro-7-cyclopropylthieno[3,2-d]pyrimidine was prepared by the method of Willis *et al.*^2^

**Scheme S2: Synthesis of 4,5-dichloro-6-methylthieno[2,3-*d*]pyrimidine (towards 2).**

4,5-Dichloro-6-methylthieno[2,3-*d*]pyrimidine was prepared by the method of Tarver Jr *et al.*^3^

**Scheme S3: Synthesis of 4-chloro-6-methylthieno[2,3-*d*]pyrimidine (towards 6).**

*Reagents and conditions*: (a) POCl_3_ (20 equiv.), 100 ^o^C, 16 h.

**Scheme S4: Synthesis of 4-chloro-5,6-dimethylthieno[2,3-*d*]pyrimidine (towards 7).**

4-Chloro-5,6-dimethylthieno[2,3-*d*]pyrimidine was purchased from Alfa Aesar [H33770].

**Scheme S5: Synthesis of 4-chloro-2,5,6-trimethylthieno[2,3-*d*]pyrimidine (towards 8).**

4-Chloro-2,5,6-trimethylthieno[2,3-*d*]pyrimidine was purchased from Enamine [EN300-07411].

**Scheme S6: Synthesis of 4-chloro-5-methyl-6-(trifluoromethyl)thieno[2,3-*d*]pyrimidine (towards 9).**

*Reagents and conditions*: (a) NaOMe (4 equiv.), 1,4-dioxane, rt, 18 h; (b) Tf_2_O (4.5 equiv.), pyridine (4.5 equiv.), [Ru(bpy)_3_]Cl_2_**.**6H_2_O (4 mol%), ClCH_2_CH_2_Cl, Blue LED light, rt, 72h; (c) conc. HCl, 70 ^o^C, 4 h; (d) POCl_3_, 100 ^o^C, 4 h.

**Scheme S7: Synthesis of 4-chloro-6-methyl-5-(trifluoromethyl)thieno[2,3-*d*]pyrimidine (towards 10).**

*Reagents and conditions*: (a) CF_3_SO_2_Na (3.0 equiv.), CF_3_CO_2_H (1.0 equiv.), CHCl_3_-H_2_O (3.5:1), tBuOOH (70 % aq., 5.0 equiv.), rt, 24 h; (b) POCl_3_, 100 ^o^C, 4 h.

**Scheme S8: Synthesis of 2-((6-cyano-5-methylthieno[2,3-*d*]pyrimidin-4-yl)thio)acetic acid (11).**

*Reagents and conditions*: (a) HSCH_2_CO_2_Me (1.2 equiv.), NEt_3_ (2.1 equiv.), MeOH, 0 ^o^C to rt; (b) NaOH (1 M) (2 equiv.), THF, 0 ^o^C, then HCl (1 M), 0 ^o^C; (c) Zn(CN)_2_ (0.66 equiv.), iPrNEt_2_ (1.05 equiv.), Xantphos-Pd-G3 (6 mol%), AcNMe_2_, 85 °C, 18 h**.**

4-Chloro-6-iodo-5-methylthieno[2,3-d]pyrimidine was purchased from Key Organics [DS-20236].

**Scheme S9: Synthesis of 4-chloro-6-cyano-7-cyclopropylthieno[3,2-*d*]pyrimidine (towards 12).**

*Reagents and conditions*: (a) LDA (4.0 equiv.), THF, -78 °C 1 h *then* **A** (4.0 equiv.), -78 °C-rt, 16 h**,** 36 %; (b) conc. HCl, 70 ^o^C, 0.25 h; (c) POCl_3_ (30 equiv.), 90 ^o^C, 2 h, 34 % over 2 steps.

**Scheme S10: Synthesis of 4-chloro-7*H*-pyrrolo[2,3-*d*]pyrimidine (towards 13).**

4-Chloro-7*H*-pyrrolo[2,3-*d*]pyrimidine was purchased from Fluorochem [043654].

**Scheme S11: Synthesis of 4,5-dichloro-7*H*-pyrrolo[2,3-*d*]pyrimidine (towards 14).**

*Reagents and conditions*: (a) NCS (1.05 equiv.), DMF, rt, 16 h.

**Scheme S12: Synthesis of 2-((7-methyl-7*H*-pyrrolo[2,3-*d*]pyrimidin-4-yl)thio)acetic acid (15).**

*Reagents and conditions*: (a) HSCH_2_CO_2_Me (1.05 equiv.), Et_3_N (2.1 equiv.), MeOH, 70 ^o^C, 16 h; (b) MeI (1.2 equiv.), NaH 60% wt on mineral oil (1.2 equiv.), 2N NaOH (2 equiv.), DMF, 0 ^o^C – rt, 2 h.

**Scheme S13: Synthesis of 2-((7-benzyl-7*H*-pyrrolo[2,3-*d*]pyrimidin-4-yl)thio)acetic acid (16).**

*Reagents and conditions*: (a) HSCH_2_CO_2_Me (1.05 equiv.), Et_3_N (2.1 equiv.), MeOH, 70 ^o^C, 16 h; (b) BnBr (1.2 equiv.), NaH 60% wt on mineral oil (1.2 equiv.), 2N NaOH (2 equiv.), DMF, 0 ^o^C – rt, 2 h.

**Scheme S14: Synthesis of 4-chloro-1,3-dimethyl-1*H*-pyrazolo[3,4-*d*]pyrimidine (towards 17).**

4-Chloro-1,3-dimethyl-1*H*-pyrazolo[3,4-*d*]pyrimidine was purchased from Key Organics [LD-0738].

**Scheme S15: Synthesis of 4-chloro-5,6-dimethylfuro[2,3-*d*]pyrimidine (towards 18).**

*Reagents and conditions*: (a) HCO_2_H (60 equiv.), 100 ^o^C, 18 h; (b) POCl_3_, 100 ^o^C, 4 h.

**Scheme S16: Synthesis of 4-chloro-5-methyl-6-(trifluoromethyl)furo[2,3-*d*]pyrimidine (towards 19).**

*Reagents and conditions*: (a) NaOMe (4 equiv.), 1,4-dioxane, rt, 18 h; (b) Tf_2_O (4.5 equiv.), pyridine (4.5 equiv.), [Ru(bpy)_3_]Cl_2_**.**6H_2_O (4 mol%), ClCH_2_CH_2_Cl, Blue LED light, rt, 72h; (c) conc. HCl, 70 ^o^C, 4 h; (d) POCl_3_, 100 ^o^C, 4 h.

**Supplementary material references**

1. Atkinson, B.N., Steadman, D., Zhao, Y., Sipthorp, J., Vecchia, L., Ruza, R.R., Jeganathan, F., Lines, G., Frew, S., Monaghan, A., Kjaer, S., Bictash, M., Jones, E.Y., Fish, P.V. Discovery of 2-phenoxyacetamides as inhibitors of the Wnt-depalmitoleating enzyme NOTUM from an X-ray fragment screen. *Med. Chem. Commun.* **2019**, *10*, 1361-1369.
2. Willis, N.J., Bayle, E.D., Papageorgiou, G., Steadman, D., Atkinson, B.A., Mahy, W., Fish, P.V. An improved, scalable synthesis of Notum inhibitor LP-922056 using 1-chloro-1,2-benziodoxol-3-one as a superior electrophilic chlorinating agent. *Beilstein Arch.* **2019**, 201970. doi:10.3762/bxiv.2019.70.v1.

3. (a) Tarver Jr, J. E., Pabba, P.K., Barbosa, J., Han, Q., Gardyan, M.W., Brommage, R., Thompson, A.Y., Schmidt, J.M., Wilson, A.G.E., He, W., Lombardo, V.K., Carson, K.G. Stimulation of cortical bone formation with thienopyrimidine based inhibitors of NOTUM Pectinacetylesterase. *Bioorg. Med. Chem. Lett.* **2016**, *26*, 1525–1528; (b) Barbosa, J., Carson, K. G., Gardyan, M. W., He, W., Lombardo, V., Pabba, P., Tarver Jr, J. Inhibitors of notum pectinacetylesterase and methods of their use. US20120065200
